# Change in public awareness of colorectal cancer symptoms following the *Be Cancer Alert Campaign* in the multi-ethnic population of Malaysia

**DOI:** 10.1186/s12885-020-06742-3

**Published:** 2020-03-25

**Authors:** Désirée Schliemann, Darishiani Paramasivam, Maznah Dahlui, Christopher R. Cardwell, Saunthari Somasundaram, Nor Saleha Binti Ibrahim Tamin, Conan Donnelly, Tin Tin Su, Michael Donnelly

**Affiliations:** 1 Centre for Public Health and UKCRC Centre of Excellence for Public Health, Queen’s University Belfast, Belfast, UK; 2 Centre for Population Health (CePH), Department of Social and Preventive Medicine, University of Malaya, Kuala Lumpur, Malaysia; 3 Facultas Public Health, University Airlangga, Surabaya, Indonesia; 4 National Cancer Society Malaysia, Kuala Lumpur, Malaysia; 5 Ministry of Health Malaysia, Kuala Lumpur, Malaysia; 6 National Cancer Registry Ireland, Cork, Ireland; 7 South East Asia Community Observatory (SEACO), Jeffrey Cheah School of Medicine and Health Sciences, Monash University Malaysia, Subang Jaya, Malaysia

**Keywords:** Colorectal cancer, Bowel cancer, Awareness, Mass media, Social media, Campaign, TV, Radio, Colonoscopy, iFOBT, Recognition, Effectiveness, Reach, Health promotion, Malaysia

## Abstract

**Background:**

Colorectal cancer (CRC) cases are detected late in Malaysia similar to most Asian countries. The *Be Cancer Alert Campaign* (BCAC) was a culturally adapted mass media campaign designed to improve CRC awareness and reduce late detection in Malaysia. The evaluation of the BCAC-CRC aimed to assess campaign reach, campaign impact and health service use.

**Methods:**

Participants aged ≥40 years (*n* = 730) from randomly selected households in Selangor State Malaysia, completed interview-based assessments. Campaign reach was assessed in terms of responses to an adapted questionnaire that was used in evaluations in other countries. The impact of the campaign was assessed in terms of awareness, confidence to detect symptoms and self-efficacy to discuss symptoms with a doctor as captured by the *Cancer Awareness Measure (CAM).* CAM was administered before-and-after campaign implementation and responses by BCAC recognisers (i.e. participants who recognised one or more of the BCAC television, radio or print advertisements when prompted) and non-recognisers (i.e. participants who did not recognise any of the BCAC advertisements) were compared analytically. Logistic regression analysed comparative differences in cancer awareness by socio-demographic characteristics and recognition of the BCAC materials.

**Results:**

Over 65% of participants (*n* = 484) recognised the BCAC-CRC. Campaign-recognisers were significantly more likely to be aware of each CRC symptom at follow-up and were more confident about noticing symptoms (46.9% vs 34.9%, *p* = 0.018) compared to non-recognisers. There was no difference between groups in terms of self-efficacy to see a doctor about symptoms. Improved symptoms awareness at follow-up was lower for Indians compared to Malays (adjusted odds ratio (OR) 0.53, 95% Confidence Interval (CI): 0.34, 0.83, *p* = 0.005). Health service use data did not indicate an increase in screening activity during or immediately after the campaign months.

**Conclusion:**

Overall, the findings of the evaluation indicated that the culturally adapted, evidence-based mass media intervention improved CRC symptom awareness among the Malaysian population; and that impact is more likely when a campaign operates a differentiated approach that matches modes of communication to the ethnic and social diversity in a population.

## Background

Colorectal cancer (CRC) is the commonest cancer in Malaysian men (age-standardised incidence rate 14.8/ 100,000), the second most common cancer in Malaysian women (age-standardised incidence rate 11.1/ 100,000) [1] and the third commonest cause of cancer deaths in Malaysia [2]. About 66% of male and 65% of female CRC cases are detected at a late stage (stage 3 or 4) thereby leading to an increased risk of cancer death. Late presentation is due, at least partly, to low cancer awareness and misbeliefs about cancer. For example, research indicates that there is a lack of awareness among Malaysians about CRC symptoms [3–5], i.e. only 40.6% of 2379 participants recognised ‘blood in stool’ as a warning sign for CRC [3]. Other causes of delayed detection and diagnosis include denial, negative perceptions of the disease, the over-reliance on traditional medicine, misperceived risk, emotional barriers and negative perceptions towards screening [6–8]. Cancer awareness campaigns and their evaluation are sparse in low- and middle-income countries (LMICs) such as Malaysia.

Collaborators from Malaysia (University of Malaya, Monash University Malaysia, National Cancer Society Malaysia (NCSM) and the Ministry of Health Malaysia (MoH)) and Queen’s University Belfast designed and implemented the *Be Cancer Alert Campaign* (BCAC) [9, 10], a culturally acceptable mass media campaign for Malaysians, based on successfully implemented campaigns in the UK [11, 12]. This research assessed the reach of the BCAC-CRC campaign as well as campaign impact, i.e. improved knowledge about CRC symptoms, perceived confidence to detect symptoms, and self-efficacy to visit a doctor to discuss CRC symptoms and health service use, i.e. number of CRC screenings undertaken (Immunochemical Faecal Occult Blood Test (iFOBT) and colonoscopies) and the number of CRC cases diagnosed.

## Methods

This was a quasi-experimental study with before- and after- evaluation assessments. The protocol for the evaluation of the BCAC-CRC was published previously [9] and it is explained here in brief.

### Study population and sampling

Malaysia is a multi-ethnic country comprising three main ethnicities: Malay (69.1%), Chinese (23%) and Indian (6.9%) [13]. The sample was drawn from Selangor State, specifically from the Rawang area because of its multi-ethnic composition [9]. Trained research assistants visited randomly selected households and invited residents to participate if they I) were aged 40 years or older, II) spoke English or Malay, III) were able to provide answers independently without support from others and IV) provided consent. Participants were interviewed 1 to 12 weeks before and 1 to 12 weeks after the BCAC-CRC was implemented.

### Intervention

The BCAC-CRC campaign was implemented over a five-week period (2nd April – 6th May 2018). A description of campaign materials was presented previously [14] and a summary is presented in Additional file 1: Table 1. Television (TV) and radio advertisements were aired nationwide and print materials (i.e. billboards, street buntings, banners, posters and brochures) were distributed throughout the study area. A social media campaign was delivered through the NCSM Facebook page. All materials contained a link to a bespoke BCAC website and the NCSM helpline.

### Data collection

#### Questionnaire

The first section of the household interview comprised questions regarding socio-demographic characteristics (e.g. gender, age, education and ethnicity), CRC history (of respondent and/or close relatives and friends), CRC screening history and monthly household income.

The second section of the interview comprised questions from the well-validated *Cancer Awareness Measure* (CAM) [5, 15] to assess campaign impact on CRC awareness as well as perceived confidence to notice symptoms and self-efficacy to discuss symptoms with a doctor. *Unprompted* knowledge about CRC signs and symptoms was assessed via the CAM by asking, ‘There are many warning signs and symptoms of CRC. Please name as many as you can think of’. *Prompted* awareness was assessed by asking, ‘Do you think [symptom] could be a sign for CRC?’ A score was calculated for unprompted and prompted awareness, respectively, by summing the ‘correct’ answers for each set of questions. In addition, confidence to recognise a CRC symptom and help-seeking was assessed via CAM questions.

A third section was included in the post-campaign household interview to assess campaign reach. This section was adapted from the *Be Cancer Aware* (BCA) campaign evaluation [16]. The questions assessed whether or not the sample I) recognised materials and II) took action as a result of the campaign. The first three questions were used to identify which TV channels, radio stations and newspapers were viewed, listened to, or read by interviewees (up to three options per type of media). Next, participants were shown the BCAC logo and other campaign materials and asked whether or not they previously noticed each item. The final set of questions asked participants whether or not they found the materials relevant, thought provoking and culturally acceptable; whether or not they shared/discussed the campaign information with their family and/or friends and whether or not they or their family and/or friends visited a health care professional as a result of seeing the BCAC-CRC campaign.

#### Social media monitoring

An external agency was hired to monitor the performance of the social media aspect of the campaign on a daily basis and to boost posts of particular interest to followers. Weekly feedback was provided to the research team regarding post reach (total number of unique users who saw the advertisement/post on their Facebook feed), interaction (total number of emoji reactions including like, love, smile, wow, sad and angry), amplification (number of shares per post), conversation (number of comments per post) and total engagement (total number of interactions, amplification and conversation per post) and recommendations were made to improve performance throughout the intervention period.

#### Helpline

The NCSM helpline was monitored by trained nurses who kept records of callers who obtained the helpline number from one of the BCAC-CRC materials. Date of call, gender of caller, reason for calling and campaign source were recorded in an Excel template (with consent from each caller).

#### Health service use

Staff in local health clinics and hospitals recorded and reported (in Excel) the number of iFOBTs and colonoscopies that were undertaken between January and July 2018 as well as information on gender, age (for iFOBT data only) and ethnicity.

### Sample size

It was estimated that 550 participants would allow 80% power to detect, as statistically significant at the 5% level, an increase by 6% in the proportion of individuals who were aware of changes in bowel habits as a symptom of CRC based upon a two sided McNemar’s Test [9].

### Data analysis

Data were analysed with SPSS vs 24. Pre- and post-campaign differences in knowledge/awareness were assessed through the McNemar test for dichotomous variables and the Wilcoxon Singed Rank test for categorical variables. Chi-square tests were conducted to test associations between campaign recognition and CRC knowledge/ awareness/attitudes; and to test associations between CRC history or CRC screening history and CRC symptoms awareness. Participants who recognised one or more BCAC-CRC materials (TV, radio or print) when prompted were referred to as ‘campaign-recognisers’ and participants who did not recognise any BCAC-CRC materials when prompted were referred to as ‘non-recognisers’. Logistic regression investigated the relationship between BCAC-CRC recognition (yes versus no) and potential explanatory variables including socio-demographic variables. The final model from which adjusted estimates were calculated contained age (in categories), gender, ethnicity, marital status, education, monthly family household income, CRC history and CRC screening history (received CRC screening – either immunochemical Faecal Occult Blood Test (iFOBT) or colonoscopy- in the past 5 years) and results are presented as odds ratios (OR) and 95% Confidence Intervals (95% CI). Similar models were applied for the outcome ‘knowledge improved’ (yes vs no). Logistic regression analyses were repeated using robust standard errors to adjust for potential clustering within households [17] (the results were similar to the results that are presented here). Service utilisation data were charted over the relevant time periods.

## Results

### Campaign fidelity

All components of the BCAC-CRC were implemented as planned and described in our pre-specified protocol and according to procedural checklists [9] (Additional file 1: Table 1).

### Study population

At baseline, 954 participants (from 710 households) completed the CRC survey of which 730/954 (from 559 households) also completed the follow-up survey (76.5%). The majority of the study population who completed the interview at both time points were female (65.1%), married (81.8%) and of Malay ethnicity (56.2%), followed by Indian (28.1%), Chinese (10%) and others (5.8%) (Table 1). ‘Others’ mainly comprised participants from Indonesian and Philippine origin. The majority of participants were followers of Islam (63%), followed by Hinduism (24%) and Buddhism (8.5%). About one third of participants were aged between 40 to 49 years (31.1%) and 50 to 59 years (36.4%). More than half of the study population attained secondary education (51.9%) or tertiary education (11.4%). According to recent government income-grouping [18], 83% of participants lived in ‘low income’ households, i.e. had a monthly family income of less than Malaysian Ringgit (RM) 4000. Significantly fewer Chinese participants, males and participants with tertiary education completed the survey at follow-up compared to baseline (Table 1). Socio-demographic characteristics by the ethnic group of participants at post-campaign assessment are presented in Additional file 1: Table 2.

**Table 1:** Socio-demographic characteristics of respondents pre- and post-campaign

	Pre n (%) *n* = 954	Post n (%) *n* = 730
**Age**		
40–49 years	314 (33.0)	227 (31.2)
50–59 years	346 (36.3)	265 (36.4)
60–69 years	216 (22.7)	177 (24.3)
≥ 70 years	76 (8.0)	59 (8.1)
**Gender**		
Males	361 (37.8)	255 (34.9)
Females	593 (62.2)	475 (65.1)
**Ethnicity**		
Malay	516 (54.1)	410 (56.2)
Chinese	110 (11.5)	73 (10.0)
Indian	264 (27.7)	205 (28.1)
Others	64 (6.7)	42 (5.8)
**Religion**		
Islam	585 (61.4)	460 (63.0)
Christianity	35 (3.7)	25 (3.4)
Buddhism	95 (10.0)	62 (8.5)
Hinduism	226 (23.7)	175 (24.0)
Others	11 (1.2)	8 (1.0)
**Marital status**		
Single^a^	167 (17.6)	133 (18.2)
Married	783 (82.4)	596 (81.8)
**Education**^**b**^		
No formal education	152 (16.0)	124 (17.0)
Primary	190 (20.0)	143 (19.6)
Secondary	485 (51.0)	378 (51.9)
Tertiary	124 (13.0)	83 (11.4)
**Family income**^**c**^		
< RM 4000	661 (81.8)	512 (83.0)
RM 4000-10,000	117 (14.5)	87 (14.1)
> RM 10,000	30 (3.7)	18 (2.9)
**CRC history**^d^		
No	833 (87.3)	633 (87.8)
Yes	112 (11.7)	88 (12.2)
**CRC screening history***(in past 5 years)*		
No	862 (90.4)	660 (90.4)
Yes	92 (9.6)	70 (9.6)

Missing variables (of participants who completed follow-up): Age (*n* = 2), Religion (*n* = 1) Marital status (*n* = 1), Education (*n* = 2), Family Income (*n* = 113), CRC history (*n* = 9)

*n* Number, *CRC* Colorectal cancer, *RM* Malaysian Ringgit

^a^ Participants who are widowed, divorced and who never married

^b^ No formal education – includes never schooled/ never completed primary school; primary education – includes completed primary school; secondary education – includes completed form 3/ completed form 5/ certificate/ A-level/ STPM/ HSC; tertiary education – includes diploma/ bachelor degree/ post-graduate degree

^c^ Monthly income of all household family members combined

^d^ CRC history includes self/ family/ friends; those who answered ‘yes’ to CRC history and CRC screening were reported as CRC history only

The most commonly viewed TV channels were the Malay channels (TV3 (55.1%), TV1 (20.5%), TV2 (19.2%) and TV9 (14.1%)). The Chinese channel (8TV) was viewed by 20.5% of Chinese participants. More than half of participants did not listen to the radio (51.8%). The most popular Malay radio stations were Sinar FM (12.7%) and Era (9.1%). The Indian stations, Thr Raaga (10.8%) and Minnal FM, were followed by 26.4 and 25.4% of Indians, respectively. Only 1% of participants reported listening to Lite FM (English station). Almost half of participants did not read newspapers (45.9%). Harian Metro was the most popular newspaper (17.2%), followed by Berita Harian (11.2%), Utusan Malaysia (11.2%) and Kosmo (8.7%).

### Campaign reach

When prompted, 26% of participants reported that they saw the BCAC logo previously. Participants reported without prompting that they noticed BCAC-CRC materials (Additional file 1: Figure 1), mainly in the form of posters that were on display in clinics (18.5%), TV advertisements (6.7%) and outdoor display boards (5.6%). When interviewees were prompted or shown the campaign materials that appeared on TV, radio and as print materials (billboards, buntings or posters), 66.3% reported that they saw at least one of the materials, particularly the TV (42.9%), print indoor/outdoor (40%) and radio announcements (18.4%) (Additional file 1: Figure 2). Approximately 71% of Malays saw at least one of the BCAC-CRC materials followed by 68% of Indians and 34% of Chinese participants. More Malays saw the TV advertisement compared to Chinese and Indians (52.9, 24.7 and 25.9%, respectively) (Additional file 1: Figure 3). Radio advertisements reached comparatively more Indians (42.9%) than Malays (10%) and Chinese (1.4%). Print displays were more effective in reaching Malays and Indians compared to Chinese (44.9, 41.4 and 17.8% respectively).

The odds that survey participants saw one or more of the BCAC materials (TV, radio and/or print) were significantly lower for Chinese interviewees compared to Malays (adjusted OR 0.23, 95% CI 0.12; 0.43, *p* < 0.001) (Table 2). Furthermore, the odds that participants saw the media campaign appeared to decrease with age and was statistically significant for those aged 70 years or older (adjusted OR comparing over 70s with 40 to 50 year olds was 0.44, 95% CI 0.21; 0.95, *p* = 0.036). Primary and secondary education completion (compared to no formal education) exerted a positive influence on campaign reach (adjusted OR 2.45, 95% CI 1.32; 4.55, *p* = 0.004 and OR 1.89, 95% CI 1.11; 3.23, *p* = 0.020, respectively).

**Table 2:** The relationship between the socio-demographic characteristics of respondents and their recognition of any aspect of the BCAC-CRC^a^

	n/d (%)	OR (95% CI) (unadjusted)	*P*	OR (95% CI) (adjusted)^b^	*P*
**Age**					
40–49 years	161/227 (70.9)	*Reference*	< 0.001	*Reference*	0.074
50–59 years	191/265 (72.1)	1.06 (0.72, 1.57)	0.778	1.11 (0.71, 1.74)	0.641
60–69 years	103/177 (58.2)	0.57 (0.38, 0.86)	0.008	0.74 (0.44, 1.25)	0.263
≥ 70 years	27/59 (45.8)	0.35 (0.19, 0.62)	< 0.001	0.44 (0.21, 0.95)	0.036
**Gender**					
Male	162/255 (63.5)	*Reference*		*Reference*	
Female	322/475 (67.8)	1.21 (0.88, 1.66)	0.246	1.13 (0.75, 1.69)	0.566
**Ethnicity**					
Malay	290/410 (70.7)	*Reference*	< 0.001	*Reference*	< 0.001
Chinese	25/73 (34.2)	0.22 (0.13, 0.37)	< 0.001	0.23 (0.12, 0.43)	< 0.001
Indian	140/205 (68.3)	0.89 (0.62, 1.28)	0.534	0.99 (0.64, 1.53)	0.975
Others	29/42 (69.0)	0.92 (0.46, 1.84)	0.820	1.00 (0.45, 2.26)	0.995
**Marital Status**					
Married	397/596 (66.6)	*Reference*		*Reference*	
Single	86/133 (64.7)	0.92 (0.62, 1.36)	0.667	1.04 (0.63, 1.73)	0.875
**Education**					
No formal education	68/124 (54.8)	*Reference*	0.024	*Reference*	0.032
Primary	100/143 (69.9)	1.92 (1.16, 3.17)	0.011	2.45 (1.32, 4.55)	0.004
Secondary	261/378 (69.0)	1.84 (1.21, 2.78)	0.004	1.89 (1.11, 3.23)	0.020
Tertiary	53/83 (63.9)	1.46 (0.82, 2.57)	0.198	2.05 (0.94, 4.44)	0.070
**Monthly family income**					
< RM 4000 (low)	357/511 (69.9)	*Reference*	0.355	*Reference*	0.296
RM 4000–10,000 (middle)	57/87 (65.5)	0.83 (0.51, 1.33)	0.433	0.80 (0.46, 1.39)	0.432
RM > 10,000 (high)	10/18 (55.6)	0.54 (0.21, 1.40)	0.207	0.46 (0.16, 1.30)	0.141
**CRC history**					
No	419/633 (66.2)	*Reference*		*Reference*	
Yes	61/88 (69.3)	1.15 (0.71, 1.87)	0.561	1.03 (0.59, 1.79)	0.915
**CRC screening history**					
No	440/660 (66.7)	*Reference*		*Reference*	
Yes	44/70 (62.9)	0.85 (0.51, 1.41)	0.522	1.24 (0.64, 2.42)	0.530

*n* number of participants ‘reached’ or who reported that they saw (one or more parts of) the campaign divided by the total number of survey participants (*d* denominator)

*BCAC* Be Cancer Alert Campaign, *CI* Confidence interval, *CRC* Colorectal cancer, *OR* Odds ratio, *RM* Malaysian Ringgit

^a^ This includes participants who reported that they have been exposed to either the TV, Radio and/or BCAC-CRC print advertisements when prompted with the advertisement at follow-up

^b^ Adjusted for age, gender, ethnicity, marital status, education, monthly family income, CRC history, CRC screening history

Participants reported the TV advertisement was most thought-provoking and relevant to them (47.7 and 55.8%, respectively), followed by the print materials (28.2 and 33.8%, respectively) and radio advertisement (14.2 and 15.9%, respectively) (Additional file 1: Figure 2). Only 2.3% reported that the advertisements were not culturally acceptable. Furthermore, 19.7% of participants replied that they, their friends or family saw a doctor as a result of seeing the advertisement (data not shown).

A total of 24 Facebook ‘posts’ were created and posted throughout the five-week campaign period (including interactive posts such as mini-quizzes to engage the target population). Most posts were posted in Malay and English and some posts were presented in Chinese and Tamil. Facebook analytics indicated that the post with the highest engagement (e.g. ‘likes’) used visuals (e.g. graphics) to explain CRC (reach 51,132; total engagement 2065). The post with the greatest reach (or number of users/viewers) contained information about the signs and symptoms of CRC (reach: 92,678; total engagement: 1493). The post with the next greatest reach described the risk factors of CRC (reach: 18,474; total engagement: 1075). Posts in Bahasa Melayu yielded the highest total engagement level whilst posts in the Indian and Chinese languages attained very limited reach and engagement.

Six calls to the NCSM Helpline were from callers who requested information regarding CRC and who mentioned that they found out about the helpline from the BCAC-CRC materials. Four of those callers heard the BCAC-CRC radio advertisement, one found out about the campaign through the website and one caller saw the Facebook advertisement.

### Campaign impact

There was a significant improvement in the recognition of all CRC symptoms (prompted) at follow up and a significant improvement in the knowledge of three unprompted symptoms, i.e. ‘blood in stool’, ‘feeling that the bowel does not empty after using the lavatory’ and ‘unexplained weight loss’ (Table 3). This pattern was reflected in overall average prompted symptom awareness (pre-campaign Mean: 4.2 (SD: 3.0) and post-campaign Mean: 5.2 (SD: 3.2); *p* < 0.001) (Additional file 1: Table 3).

**Table 3:** Colorectal cancer awareness pre- and post-campaign (*n* = 730) and between BCAC-CRC recognisers and non-recognisers

**Survey question**	**Pre** **n (%)**	**Post** **n (%)**	***P(McNemar)***	**Knowledge improvement in BCAC recognisers** **n (%)**^**a**^	**Knowledge improvement in BCAC non-recognisers** **n (%)**^**b**^	***P*** ***(Chi -Square)***
**Signs and symptoms (unprompted)**						
Blood in stool	33 (4.5)	142 (19.5)	< 0.001	83/460 (18.0)	38/237 (16.0)	0.577
Persistent abdominal pain	165 (22.6)	186 (25.5)	0.150	84/359 (23.4)	23/206 (11.2)	0.001
Change in bowel habits for several weeks	69 (9.5)	88 (12.1)	0.102	55/432 (12.7)	15/229 (6.6)	0.020
Feeling that bowel does not empty after using lavatory	30 (4.1)	48 (6.6)	0.034	30/461 (6.5)	11/239 (4.6)	0.396
Pain in back passage	1 (0.1)	6 (0.8)	0.125	4/483 (0.8)	2/246 (0.8)	0.999
Bleeding from back passage	26 (3.6)	14 (1.9)	0.074	13/465 (2.8)	0/239 (0.0)	0.006
Tiredness/ anaemia	16 (2.2)	25 (3.4)	0.176	17/474 (3.6)	5/240 (2.1)	0.385
Unexplained weight loss	10 (1.4)	33 (4.5)	0.001	26/476 (5.5)	6/244 (2.5)	0.097
Lump in your abdomen	3 (0.4)	9 (1.2)	0.146	6/481 (1.2)	3/246 (1.2)	0.999
**Signs and symptoms (prompted)**						
Blood in stool	394 (54.0)	492 (67.4)	< 0.001	123/208 (59.1)	55/128 (43.0)	0.006
Persistent abdominal pain	372 (51.0)	441 (60.4)	< 0.001	117/212 (55.2)	49/146 (33.6)	< 0.001
Change in bowel habits for several weeks	335 (45.9)	403 (55.2)	< 0.001	128/253 (50.6)	43/142 (30.3)	< 0.001
Feeling that bowel does not empty after using lavatory	330 (45.2)	396 (54.2)	< 0.001	124/248 (50.0)	50/152 (32.9)	0.001
Pain in back passage	256 (35.1)	384 (52.6)	< 0.001	161/302 (53.3)	53/172 (30.8)	< 0.001
Bleeding from back passage	339 (46.4)	446 (61.1)	< 0.001	141/255 (55.3)	51/136 (29.7)	0.001
Tiredness/ anaemia	283 (38.8)	379 (51.9)	< 0.001	137/275 (49.8)	54/172 (31.4)	< 0.001
Unexplained weight loss	378 (51.8)	415 (56.8)	0.031	109/220 (49.5)	48/132 (36.4)	0.022
Lump in your abdomen	358 (49.0)	410 (56.2)	0.003	116/221 (52.5)	57/151 (37.7)	0.007
**Attitudes**	**Pre** **n (%)**	**Post** **n (%)**	***P*** ***(Mc Nemar)***	**Attitude improvement in BCAC recognisers** **n (%)**	**Attitude improvement in BCAC non-recognisers** **n (%)**	***P*** ***(Chi-square)***
How confident are you that you would notice a CRC sign or symptom? *(Those ‘very confident’ or ‘fairly confident’)*	223 (33.2)	290 (39.7)	< 0.001	145/309 (46.9)	53/152 (34.9)	0.018
How soon would you go and see a doctor if you noticed a CRC sign/symptom? *(Those who replied < 2 weeks.)*	665 (91.1)	678 (92.9)	0.608	34/38 (89.5)	20/22 (90.9)	0.999

Missing information (for participants who completed follow up only): Prompted symptoms (*n* = 1); Confidence (*n* = 110), delayed help seeking (*n* = 70)

*BCAC* Be Cancer Alert Campaign, *CRC* Colorectal Cancer, *n* Number of participants

^a^ Number of participants who recognised the BCAC and did not know the CRC symptom at baseline but knew the symptom at follow up, divided by the total number of participants who recognised the campaign and did not know the CRC symptom at baseline

^b^ Number of participants who did not recognise the BCAC and did not know the CRC symptom at baseline but know the symptom at follow up, divided by the total number of participants who did not recognise the campaign and did not know the CRC symptom at baseline

Regarding participants who were not aware of CRC symptoms at baseline, a significantly higher proportion of BCAC recognisers compared to BCAC non-recognisers improved their awareness at follow-up for each prompted CRC symptom (Table 3). Similarly, change in average symptom awareness scores was higher for BCAC recognisers than non-recognisers (BCAC recognisers Mean: 1.2 (SD: 3.5) vs. BCAC non-recognisers Mean: 0.6 (SD: 3.3); *p* = 0.014) (Additional file 1: Table 3). Unprompted knowledge about particular CRC symptoms at follow-up was significantly higher among BCAC recognisers who did not know the symptoms at baseline compared to non-recognisers for the following symptoms: ‘persistent abdominal pain’ (23.4% vs 11.2%, *p* = 0.001, respectively), ‘change in bowel habits for several weeks’ (12.7% vs 6.6%, *p* = 0.020, respectively) and ‘bleeding from back passage’ (2.8% vs 0%, *p* = 0.021, respectively).

Confidence in recognising a CRC symptom (fairly or very confident) increased significantly at follow-up (33.2% vs 39.7%, *p* < 0.001). A higher proportion of BCAC-CRC recognisers who were not confident at baseline compared to non-recognisers who were not confident at baseline, reported at follow-up that they were confident about symptom recognition (46.9% vs 34.9%, *p* = 0.018) (Table 3). Most participants at baseline (91.1%) and at follow-up (92.9%) reported that they would visit a doctor within 2 weeks if they noticed a CRC sign/symptom; there was no difference between BCAC recognisers and non-recognisers.

The only variables that were significantly associated with an increase in the proportion of participants who reported awareness of, or endorsed, prompted CRC symptoms at follow-up were ethnicity and recognition of having heard or seen the radio or poster advertisement (Table 4). Being of Indian ethnicity compared to Malay was associated with significantly lower odds of having improved symptom awareness post-campaign compared to pre-campaign in the unadjusted and adjusted models (adjusted OR 0.53, 95% CI 0.34; 0.83, *p* = 0.005). There was a higher likelihood of observing an increase in symptom endorsement at follow-up among participants who heard the BCAC-CRC radio advertisement compared to participants who did not hear it (adjusted OR 2.19, 95% CI 1.33; 3.62, *p* = 0.002). Similarly, an increase in symptom endorsement or awareness at follow-up was significantly more likely among participants who saw the print advertisement (adjusted OR 1.80, 95% CI 1.27; 2.56, *p* = 0.001). TV advertisement viewing was not associated with increased CRC symptoms endorsement at follow-up.

**Table 4:** Improvement in prompted symptom awareness by socio-demographic characteristics and recognition of BCAC-CRC advertisements (binary logistic regression)

	n/d (%)	OR (95% CI) (unadjusted)	*P*	OR (95% CI) (adjusted)^a^	*P*
**Age**					
40–49 years	137/227 (60.4)	*Reference*	0.308	*Reference*	0.075
50–59 years	138/265 (52.1)	0.71 (0.50, 1.02)	0.066	0.71 (0.47, 1.06)	0.095
60–69 years	101/176 (57.4)	0.89 (0.59, 1.32)	0.548	1.26 (0.76, 2.09)	0.369
≥ 70 years	32/59 (54.2)	0.78 (0.44, 1.39)	0.395	1.19 (0.56, 2.50)	0.652
**Gender**					
Male	140/ 255 (54.9)	*Reference*		*Reference*	
Female	269/475 (56.6)	1.08 (0.79, 1.46)	0.631	1.21 (0.83, 1.77)	0.326
**Ethnicity**					
Malay	241/410 (58.8)	*Reference*	0.169	*Reference*	0.031
Chinese	42/73 (57.5)	0.95 (0.57, 1.57)	0.842	1.07 (0.58, 1.97)	0.841
Indian	101/204 (49.5)	0.69 (0.49, 0.96)	0.030	0.53 (0.34, 0.83)	0.005
Others	25/42 (59.5)	1.03 (0.54, 1.87)	0.926	1.03 (0.49, 2.17)	0.937
**Marital Status**					
Married	332/596 (55.8)	*Reference*		*Reference*	
Single	77/133 (57.9)	1.09 (0.75, 1.59)	0.660	1.12 (0.70, 1.81)	0.639
**Education**					
No formal education	69/123 (56.1)	*Reference*	0.208	*Reference*	0.135
Primary	71/143 (49.7)	0.77 (0.48, 1.25)	0.294	0.66 (0.37, 1.18)	0.162
Secondary	224/378 (59.3)	1.14 (0.76, 1.72)	0.537	1.11 (0.66, 1.87)	0.701
Tertiary	43/83 (51.8)	0.84 (0.48, 1.47)	0.544	0.81 (0.39, 1.68)	0.562
**Monthly family income**					
< RM 4000	293/511 (57.3)	*Reference*	0.558	*Reference*	0.627
RM 4000–10,000	49/87 (56.3)	0.96 (0.61, 1.52)	0.859	0.94 (0.56, 1.57)	0.798
RM > 10,000	8/18 (44.4)	0.60 (0.23, 1.53)	0.282	0.60 (0.21, 1.70)	0.336
**CRC history**					
No	356/633 (56.2)	*Reference*		*Reference*	
Yes	48/88 (54.5)	0.93 (0.60, 1.46)	0.764	0.83 (0.50, 1.38)	0.468
**CRC screening history**					
No	378/660 (57.3)	*Reference*		*Reference*	
Yes	31/69 (44.9)	0.61 (0.37, 1.00)	0.051	0.68 (0.37, 1.24)	0.206
**TV ad recognition**					
No	229/417 (54.9)	*Reference*		*Reference*	
Yes	180/312 (57.7)	1.12 (0.83, 1.51)	0.455	0.80 (0.56, 1.15)	0.232
**Radio ad recognition**					
No	322/596 (54.0)	*Reference*		*Reference*	
Yes	87/133 (65.4)	1.61 (1.09, 2.38)	0.017	2.19 (1.33, 3.62)	0.002
**Print ad recognition**					
No	223/437 (51.0)	*Reference*		*Reference*	
Yes	186/292 (63.7)	1.68 (1.24, 2.28)	0.001	1.80 (1.27, 2.56)	0.001

*n* number of participants improved their prompted symptom awareness by one or more symptoms divided by the total number of survey participants (*d* denominator)

*Ad* Advertisement, *CI* Confidence interval, *n* Number of participants, *OR* Odds ratio, *RM* Malaysian Ringgit, *TV* Television

^a^ Adjusted for age, gender, ethnicity, marital status, education, monthly family income, TV ad recognition, radio ad recognition, print ad recognition, CRC history, CRC screening

### Health service use

**Fig. 1:**
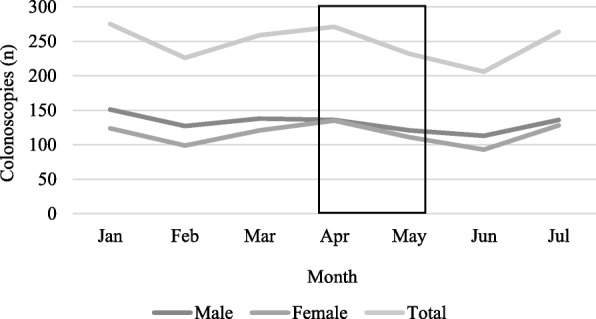
Colonoscopies in Sg Buloh and Selayang hospital by gender between January and July 2018

**Fig. 2:**
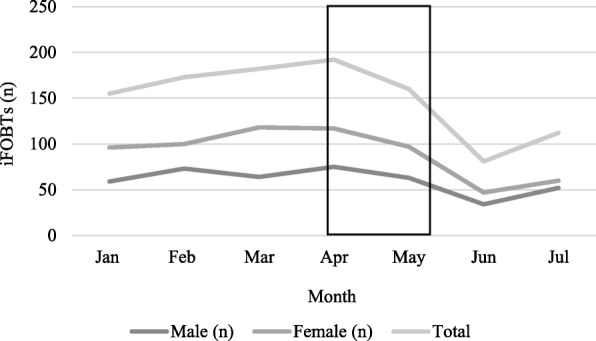
iFOBTs undertaken at clinics and hospitals between January and July 2018

Over the 7 months, 1055 iFOBTs and 1733 colonoscopies were reported by the local hospitals and clinics in the study area. Most colonoscopies were conducted in January 2018 (*n* = 275) followed by April (*n* = 271) and July (*n* = 264) (Fig. 1, Additional file 1: Table 4) and most iFOBTs were conducted in April (*n* = 192), which indicated a very small, non-significant increase compared to previous months (Fig. 2, Additional file 1: Table 4). The majority of iFOBTs (60%) and colonoscopies (53%) were conducted in males and experienced by Malays (48.9 and 47%, respectively), followed by Chinese (28.2 and 36.6%) and Indians (17.1 and 13.4%) (Additional file 1: Table 5). Data on age was provided in full for iFOBTs only: 50–59 years (22.2%), 60–69 years (24.8%) and 70 years and older (25.1%) (Additional file 1: Table 6). Staff in the clinics were unable to provide data about the number of participants who discussed CRC-related symptoms with their doctors or the number of CRC cases diagnosed.

## Discussion

Malaysians with cancer tend to present to cancer services in the later stages of the disease, and this late presentation has severe, often fatal, consequences. Therefore, increasing awareness about cancer signs and symptoms could contribute to earlier presentation and improvements in cancer outcomes. Despite numerous studies describing low CRC awareness amongst Malaysians, this was the first study that developed and evaluated a public health intervention in the form of a mass media campaign that aimed to improve CRC awareness. Generally, the results appeared to indicate low awareness about CRC signs and symptoms pre-campaign including prompted symptoms (ranging from 35 to 54% for different symptoms) and confirmed the need to design and implement ways in which to improve cancer awareness and nurture preventative efforts and early presentation. For example, pre-campaign awareness level about ‘blood in stool’ for the English *Be Clear on Cancer (BCOC)* was 55% compared to 46% in Malaysia [19]. The results of the evaluation, overall, indicated that symptom awareness improved after campaign delivery and that, more specifically, prompted awareness about all CRC symptoms improved among participants who saw any of the BCAC-CRC materials and did not recognise the symptoms as baseline, compared to participants who did not recall seeing or hearing the campaign.

This post-campaign increase in awareness may be related to the way in which the campaign materials were adapted and presented [14] and informed by best available evidence [10]. For example, print advertisements that highlighted the colon/rectum and the radio advertisements that emphasised paying attention to bowel habits were adapted to suit the multi-ethnic population and culture of Malaysia. ‘Blood in stool’ was the main symptom that was highlighted in TV and radio advertisements. Approximately 60% of BCAC-recognisers compared to 40% of non-recognisers who were unaware of this symptom at baseline reported after the campaign that blood in stool was a key important sign of CRC. Findings from the English *BCOC* four-month campaign reported a smaller increase in awareness about ‘blood in stool’, i.e. 14% post-campaign, though data comparing improvement between BCAC recognisers and non-recognisers was not reported [19].

Posters in clinics and TV advertisements were the two most commonly recognised (unprompted) media before participants were shown the three advertisements, which is in line with findings from the BCA primer and lung cancer campaigns [16]. Sixty-six percent of the study population reported that they saw one or more BCAC-CRC advertisements compared to about 70% who noticed any BCOC materials [20]. Recognition of TV advertisements was higher in the BCOC campaign (7 out of 10) compared to BCAC-CRC (5 out of 10) [20]. This result may suggest that a similar reach can be achieved with a mass media campaign of a shorter duration. Mass media campaigns do not appear to reach older participants, perhaps, because people aged over 60 years old feature rarely in such campaigns [21] including the BCAC. The BCAC found it a challenge to recruit older survivors to share their stories on TV or online. Findings from our evaluation and the BCOC campaign indicate that participants aged 75 years or above were significantly less likely to notice advertisements [20]. In contrast to the findings relating to older people, female participants in our evaluation and in the BCOC survey were more likely than men to notice advertisements. Findings from a relatively small cross-sectional USA study that aimed to assess whether or not years of CRC campaign activities including the Centre for Disease Control Prevention’s *Screen for Life campaign* improved awareness about campaign-related messages, did not find a significant difference between participants aged below or above 65 years [22].

Findings from the evaluation of the Northern Irish BCA primer campaign indicated that the extent of the ‘reach’ to lower socio-economic groups was relatively poor [16]. Whilst the BCAC-CRC was noticed least by participants without formal education, it reached participants from low-income households equally as participants from middle- or high-income backgrounds. Regarding coverage of ethnic groupings, the BCAC-CRC seemed to reach Malays and Indians but not Chinese participants despite the fact that the TV advertisement was aired for 5 weeks on one of the most commonly watched Chinese TV channels (8TV). Poor reach may be related to the lower proportion of Chinese participants who agreed to participate in the surveys and may suggest that there is a need to consider alternative ways of communicating cancer education messages to the Chinese community in Malaysia. Indeed, there may be merit in tailoring media modes to particular ethnic groups. For example, a much higher proportion of Indians than other ethnic groups listened to (Tamil) radio. Print advertisements and TV seemed to reach a similar proportion of the target population. However, viewing the TV advertisements did not affect prompted awareness about CRC signs and symptoms whereas observing or listening to printed or radio materials seemed to contribute to increased awareness. Although campaign reach to Chinese participants was low, Indians were significantly less likely to show improved CRC symptom recognition (prompted). Income and educational level groupings achieved similar awareness improvement (scores) in keeping with findings from the BCOC [12, 19].

More than half the sample thought that the BCAC TV campaign materials were relevant to them (56%), which is similar to findings from the BCOC bowel campaign (51%) [19]. Eighty-four percent of participants did not reply or answered ‘don’t know’ to the question regarding whether or not the radio materials were relevant to them. Participants who did not think that the radio campaign was relevant tended to be older (60 and above) whilst a higher proportion of Indians than other ethnic groups thought it was relevant. The poor reach to older age groups might be related to the use of unfamiliar languages e.g. English or Tamil. There were no differences between participants who viewed print advertisements as relevant vs irrelevant.

The collection of data on screening activity before and after the BCAC-CRC in a way that would have afforded a robust test of campaign impact was not possible in the circumstances. The limited screening data that we were able to collect did not indicate an increase - iFOBT and colonoscopy rates were similarly high in January and July. The results of CRC awareness raising studies in Japan, Korea and Israel (through mailed information, i.e. brochures and/or letters) were inconclusive [10] whereas findings from the more extensive BCOC media campaign indicated that the number of (2-week-wait) referrals for screening increased by 59% [23] and the Australian National Bowel Cancer Screening Programme which promoted iFOBT uptake through TV advertisements for 8 weeks reported an improvement in screening uptake during the campaign and up to 2 months after [24]. So, it appears that a multi-mode approach is needed for awareness-raising campaigns to achieve impact in relation to screening activity and clinic visits. It may not be surprising that, overall, the number of iFOBTs was higher for women whereas the number of colonoscopies was higher for men, given that CRC is more common among men. Similarly, iFOBT completion was highest among people aged 50 years and older, which is unsurprising given the higher CRC incidence in that age group and current opportunistic screening recommendations. The pattern of screening activity appeared to indicate the need to be mindful of socio-cultural contexts when designing and implementing this kind of public health intervention. For example, fewer iFOBTs and colonoscopies were undertaken during February, May and June due, in part, to the national holidays in Malaysia that occurred during these months and the observance by Muslims to avoid examination of certain bodily cavities during Ramadan’s fasting months, May – June 2018.

The fact that use of social media as part of the campaign indicated, for example, high engagement (in terms of the frequency of ‘posts’) and, at the same time, low recall of campaign posts (on Facebook) points to the difficulty of evaluating the impact of this particular intervention component. The benefits of social media have been described as widening information access and increasing information sharing and interaction [25]. However, these benefits and the diffuse and widely distributed nature of social media means that it is likely that more than the usual research techniques are required to capture its impact for public health good and cancer education at a population level. Further research is required to investigate the use and impact of social media interventions (delivered through Facebook, YouTube and other channels) in terms of delivering effective education and improving cancer awareness [26]. Regarding the helpline, there do not appear to be any studies that report the use of a helpline and its uptake as part of a cancer awareness campaign. The low number of calls to the helpline in this campaign may indicate that participants did not perceive a pressing need to call and/or preferred to visit their doctor to discuss health issues. Qualitative findings regarding the use of cancer council helplines in Australia also suggested that barriers to calling included not needing/wanting help [27]. Nevertheless, a helpline of this kind serves as an extra ‘safety net’ to capture urgent concerns from research participants.

It was not possible to create or construct a control group as part of this evaluation due to the nationwide distribution of the cancer awareness-raising intervention via TV, radio, print and social media. ‘*The major strength of mass media [as a public health and cancer education intervention] - their ability to reach a wide audience, paradoxically, also presents the greatest challenge for evaluation*’ [28]. In addition, it is possible that the pre-campaign assessment itself provided a form of cancer education about CRC symptoms or prompted participants to search out further information about CRC even though participants at baseline were not told about the campaign or that there would be a follow-up assessment. However, data about campaign recognition (or not) was used to adjust the analysis in a way that illuminated any extra effect due to the campaign. The self-reported nature of assessing campaign recognition is a commonly recognised limitation of evaluations of the kind presented here. It is important to be aware that the follow-up survey occurred between 1 day and 3 months post-campaign and, therefore, participants who were interviewed 1 month after the campaign ended may have had higher symptom awareness compared to participants who were interviewed 2 to 3 months post-campaign. Also, there is a possibility that some participants may have answered interview assessment questions in a self-perceived socially desirable way. We need to be mindful, too, of the composition of the study population in terms of, for example, the comparative underrepresentation of men [13] which was due, most likely, to the fact that research assistants visited households during the daytime when more women may have been at home. Chinese participants were also underrepresented whilst, as a proportion of the study population relative to the general population of Malaysia, there were around four times more Indian participants [13]. A strength of the evaluation was that most participants were recruited from low-income households, a section of the population who tend to be underrepresented in research. Finally, we were unable to provide data about screening services provided by privately run clinics; and we collected data with difficulty about the activity of government-funded clinics, which kept only limited paper-based records. This kind of data management and related research is common in LMICs and, so, it is an area that deserves attention and resources.

## Conclusion

Arguably, the BCAC-CRC study is one of the most robust evaluations of public health efforts to improve early cancer detection in an Asian country, particularly in the form of a cancer mass media campaign, despite the limitations that we have noted above [10]. Overall, the findings of the evaluation indicate that a culturally adapted, evidence-based mass media intervention [14] appears to impact positively in terms of improving CRC symptom awareness among an Asian population; and that impact is more likely when a campaign operates a differentiated approach that matches modes of communication to the ethnic and religious diversity in a population. Therefore, further research is needed to identify which communication channels and form of tailoring are required to reach (in the example of Malaysia) the Chinese community, people without formal education and older people. The campaign that is presented here and its evaluation provides a sound design template and research platform for the implementation and spread of cancer awareness programmes in Malaysia and Asia and, so, reduce late presentation and CRC diagnosis in Malaysia and other Asian countries. Furthermore, our partnership approach to the design of the programme including the ongoing active involvement of the MoH and the NCSM increases the likelihood of effective knowledge transfer.

## Supplementary information

**Additional file 1: Table 1.** Information about all campaign activities and media used during BCAC-CRC. **Table 2.** Socio-demographic characteristics of post-campaign respondents by ethnic group. **Table 3.** Change in average prompted knowledge score. **Table 4.** Number of iFOBTs and colonoscopies undertaken by gender (January – July 2018). **Table 5.** Number of iFOBTs and colonoscopies undertaken by ethnicity (January – July 2018). **Table 6.** Number of iFOBTs undertaken by age group (January – July 2018). **Figure 1.** Advertisement channels through which participants noticed the BCAC-CRC advertisements (unprompted). **Figure 2.** Advertisement channels through which participants noticed the BCAC-CRC advertisements (prompted) and thoughts on materials. **Figure 3.** Difference in campaign material reach between ethnicities.

## Data Availability

The datasets used and/or analysed during the current study are available from the corresponding author on reasonable request.

## Abbreviations

BCA: Be Cancer Aware
BCAC: Be Cancer Alert Campaign
BCOC: Be Clear on Cancer
CAM: Cancer Awareness Measure
CI: Confidence Interval
CRC: Colorectal cancer
iFOBT: Immunochemical Fecal Occult Blood Test
LMICs: Low Middle Income Countries
MoH: Ministry of Health
N: Number
NCSM: National Cancer Society
OR: Odds ratio
RM: Malaysian Ringgit
TV: Television
