# Supplementary material for: Change in public awareness of colorectal cancer symptoms following the Be Cancer Alert Campaign in the multi-ethnic population of Malaysia
Source: BMC Cancer. 2020 Mar 25;20:252. doi: 10.1186/s12885-020-06742-3 (PMC7093961; doi:10.1186/s12885-020-06742-3)
Supplement: Supplementary file 1 — Additional file 1: Table 1. Information about all campaign activities and media used during BCAC-CRC. Table 2. Socio-demographic characteristics of post-campaign respondents by ethnic group. Table 3. Change in average prompted knowledge score. Table 4. Number of iFOBTs and colonoscopies undertaken by gender (January – July 2018). Table 5. Number of iFOBTs and colonoscopies undertaken by ethnicity (January – July 2018). Table 6. Number of iFOBTs undertaken by age group (January – July 2018). Figure 1. Advertisement channels through which participants noticed the BCAC-CRC advertisements (unprompted). Figure 2. Advertisement channels through which participants noticed the BCAC-CRC advertisements (prompted) and thoughts on materials. Figure 3. Difference in campaign material reach between ethnicities. [file 12885_2020_6742_MOESM1_ESM.docx]

**Additional file 1**

**Table 1** Information about all campaign activities and media used during BCAC-CRC

| **Time period** (2018) | **Media (Language)** | **Content** | **Implementation area** |
| --- | --- | --- | --- |
| **TV** |  |  |  |
| 2^nd^ April – 6^th^ May  5 weeks  4-6 ads/ day | Advertisement, 30 seconds on TV3 (BM), TV9 (BM), 8TV (MAN) and YouTube (BM, MAN, ENG) | Cancer survivor highlighting the signs and symptoms he experienced, the importance of family support, that CRC affects males and females and that early detection can save lives | Nationwide |
| 8^th^ April  2 x 2 min slot (8pm news) | Prime time slot on TV3 (BM) and 8TV (MAN) | Highlighting the BCAC launch, CRC signs and symptoms and that early detection can save lives | Nationwide |
| **Radio** |  |  |  |
| 9^th^ – 22^nd^ April  2 weeks  5-7 ads/ day | Advertisement, 30 seconds on I) Thr Raaga (TAM) and II) Lite FM (ENG) | Cancer survivors highlighting CRC signs and symptoms, that CRC can be cured, that early detection can save lives and to see doctor urgently if symptoms are noticed | I) Peninsular Malaysia  II) Central Malaysia (including Rawang) |
| 3^rd^ May | Radio Interview with KL FM (ENG) | Campaign launch, CRC signs and symptoms, risk factors/ prevention of CRC |  |
| **Print** |  |  |  |
| 2^nd^ April – 6^th^ May  5 weeks | Billboards (x2) (BM) | Displayed the colon as well as the headline ‘Don’t be shy to check your stool’ and the slogan ‘Colorectal cancer can be cured if found and treated early’ | Rawang ^a^ |
|  | Banners (x15) (BM) | *See billboards* | Residential areas in Rawang ^a^  Tesco in Rawang ^a^ |
|  | Street buntings (x100) (BM) | *See billboards* | Rawang ^a^ |
|  | Posters (BM and ENG) | *See billboards +* signs and symptoms of CRC, see a doctor urgently if symptoms are noticed, prevalence of CRC in Malaysia, age-related risk, early detection can save lives, CRC can be cured | Clinics in Rawang ^a^ |
|  | Brochures (BM, ENG, MAN and TAM) | *See billboards +* what is CRC, signs and symptoms of CRC, risk factors and prevention of CRC, cancer survivor story, CRC can be cured, early detection can save lives | Clinics in Rawang ^a^ |
| 14^th^ April (x3), 16^th^ April (x1), 19^th^ April (x1) | 5x Newspaper articles (BM and ENG), i.e. Berita Harian, The Star, Kosmo, The Star (Metro South and East), Harian Metro | *Key messages same as posters* | Respective areas of coverage |
| **Social media** |  |  |  |
|  | 24 posts, including 4 boosted posts (BM, ENG, MAN, TAM)  2 x 2 post from Malaysian influencers Fara Fauzana & Jack Lim | What is CRC, BCAC-CRC campaign launch, signs and symptoms, risk factors, prevention, campaign video, cancer survivor stories (x3), call to action, early detection saves lives, quiz | Nationwide |
| **Website** |  |  |  |
|  | Link: [www.becanceralert.com](http://www.becanceralert.com) (BM and ENG) | Information about cancer in general, signs and symptoms, risk factors and early detection as well as access to all BCAC-CRC materials, cancer survivor stories | Nationwide |
| **Launch event** |  |  |  |
| 5^th^ April | 1 hour launch event (ENG) | Stakeholders and media were invited to officially launch the BCAC-CRC | University of Malaya, Kuala Lumpur |

*All materials included information about the website and NCSM hotline.*

^a^ Rawang was the study area for BCAC-CRC

Ads – advertisement, BCAC – Be Cancer Alert Campaign, BM – Bahasa Melayu, CRC – colorectal cancer, ENG- English, MAN – Mandarin, NCSM – National Cancer Society Malaysia, TAM- Tamil

**Table 2** Socio-demographic characteristics of post-campaign respondents by ethnic group

|  | **Malay** (n=410)  n (%) | **Chinese** (n=73)  n (%) | **Indian** (n=205)  n (%) | **Others** (n=42)  n (%) |
| --- | --- | --- | --- | --- |
| **Age** |  |  |  |  |
| 40-49 years | 127 (31.1) | 13 (17.8) | 63 (30.9) | 24 (57.1) |
| 50-59 years | 158 (38.6) | 16 (21.9) | 76 (37.3) | 15 (35.7) |
| 60-69 years | 94 (23.0) | 30 (41.1) | 50 (24.5) | 3 (7.1) |
| ≥70 years | 30 (7.3) | 14 (19.2) | 15 (7.4) | 0 (0) |
| **Gender** |  |  |  |  |
| Males | 156 (38.0) | 25 (34.2) | 63 (30.7) | 11 (26.2) |
| Females | 254 (62.0) | 48 (65.8) | 142 (69.3) | 31 (73.8) |
| **Religion** |  |  |  |  |
| Islam | 410 (100) | 3 (4.1) | 8 (3.9) | 39 (92.9) |
| Christianity | 0 (0) | 6 (8.2) | 18 (8.7) | 1 (2.4) |
| Buddhism | 0 (0) | 59 (80.8) | 1 (0.5) | 2 (4.8) |
| Hinduism | 0 (0) | 1 (1.4) | 174 (84.9) | 0 (0) |
| Others | 0 (0) | 4 (5.5) | 4 (2.0) | 0 (0) |
| **Marital status** |  |  |  |  |
| Single^a^ | 75 (18.3) | 12 (16.4) | 39 (19.1) | 7 (16.7) |
| Married | 335 (81.7) | 61 (83.6) | 165 (80.9) | 35 (83.3) |
| **Education^b^** |  |  |  |  |
| No formal education | 42 (10.3) | 13 (17.8) | 57 (27.8) | 12 (28.6) |
| Primary | 70 (17.2) | 19 (26.0) | 43 (21.0) | 11 (26.2) |
| Secondary | 233 (57.1) | 35 (47.9) | 94 (45.9) | 16 (38.1) |
| Tertiary | 63 (15.4) | 6 (8.2) | 11 (5.4) | 3 (7.1) |
| **Family income^c^** |  |  |  |  |
| <RM 4,000 | 271 (79.0) | 45 (78.9) | 161 (89.4) | 35 (94.6) |
| RM 4,000-10,000 | 61 (17.8) | 11 (19.3) | 14 (7.8) | 1 (2.7) |
| >RM 10,000 | 11 (3.2) | 1 (1.8) | 5 (2.8) | 1 (2.7) |
| **CRC history** ^d^ |  |  |  |  |
| Yes | 68 (16.6) | 7 (9.6) | 22 (10.7) | 1 (2.4) |
| **CRC screening history** *(in past 5 years)* |  |  |  |  |
| Yes | 29 (7.1) | 9 (12.3) | 31 (15.1) | 1 (2.4) |

n- number, RM – Malaysian Ringgit,

^a^ Participants who are widowed, divorced and who never married

^b^ No formal education – includes never schooled/ never completed primary school; primary education – includes completed primary school; secondary education – includes completed form 3/ completed form 5/ certificate/ A-level/ STPM/ HSC; tertiary education – includes diploma/ bachelor degree/ post-graduate degree

^c^ Monthly income of all household family members combined

^d^ CRC history includes self/ family/ friends; those who answered ‘yes’ to CRC history and CRC screening were reported as CRC history only

Missing variables (of participants who completed follow-up): Age (n=2), Religion (n=1) Marital status (n=1), Education (n=2), Family Income (n=113), CRC history (n=9)

**Table 3** Change in average prompted knowledge score

|  | **Pre**  Mean (SD) | **Post**  Mean (SD) | **Change**  Mean (SD) | **p-value**  *(paired sample t-test)* | **p-value** *(independent sample t-test)* |
| --- | --- | --- | --- | --- | --- |
| All participants | 4.2 (3.0) | 5.2 (3.2) | 1.0 (3.4) | <0.001 | - |
| CRC history *(self/ family/ friends)* | 5.2 (2.8) | 6.0 (2.8) | 0.9 (3.0) | 0.008 | *Comparing CRC history to no history*  0.713 |
| No CRC history *(self/ family/ friends)* | 4.1 (3.0) | 5.1 (3.3) | 1.0 (3.5) | <0.001 |  |
| CRC screening history *(self)* | 4.1 (3.3) | 4.6 (3.5) | 0.6 (3.7) | 0.196 | *Comparing CRC screening history to no history*  0.304 |
| No CRC screening history *(self)* | 4.2 (2.9) | 5.2 (3.2) | 1.0 (3.4) | <0.001 |  |
| BCAC recognisers | 4.5 (2.9) | 5.7 (3.1) | 1.2 (3.5) | <0.001 | *Comparing BCAC recognisers to non-recognisers*  0.014 |
| BCAC non-recognisers | 3.6 (3.0) | 4.1 (3.2) | 0.6 (3.3) | 0.010 |  |

**Table 4** Number of iFOBTs and colonoscopies undertaken by gender (January – July 2018)

|  | iFOBTs | | Colonoscopies | |
| --- | --- | --- | --- | --- |
|  | Males (n) | Females (n) | Males (n) | Females (n) |
| January | 59 | 96 | 151 | 124 |
| February | 73 | 100 | 127 | 99 |
| March | 64 | 118 | 138 | 121 |
| April | 75 | 117 | 136 | 135 |
| May | 63 | 97 | 121 | 111 |
| June | 34 | 47 | 113 | 93 |
| July | 52 | 60 | 136 | 128 |

**Table 5** Number of iFOBTs and colonoscopies undertaken by ethnicity (January – July 2018)

|  | iFOBTs  n=1055 n (%) | Colonoscopies  n=1733 n (%) |
| --- | --- | --- |
| Malay | 516 (48.9) | 815 (47.0) |
| Chinese | 297 (28.2) | 634 (36.6) |
| Indian | 180 (17.1) | 233 (13.4) |
| Other | 62 (5.9) | 51 (2.9) |

**Table 6** Number of iFOBTs undertaken by age group (January – July 2018)

| Age group | n (%) |
| --- | --- |
| < 40 years | 180 (17.1) |
| 40-49 years | 114 (10.8) |
| 50-59 years | 234 (22.2) |
| 60-69 years | 262 (24.8) |
| 70 years and above | 265 (25.1) |

**Figure 1** Advertisement channels through which participants noticed the BCAC-CRC advertisements (unprompted)

**Figure 2** Advertisement channels through which participants noticed the BCAC-CRC advertisements (prompted) and thoughts on materials

*relevant – this includes participants saw the materials at the survey for the first time during the post-survey and thought they were relevant to them

**Figure 3** Difference in campaign material reach between ethnicities
